# A randomized controlled trial of social media promotion in neurosurgical publishing

**DOI:** 10.1007/s10143-024-02597-5

**Published:** 2024-07-27

**Authors:** Moira Vieli, Bianca Battilana, Alex Alamri, Katrin Rabiei, Laura Lippa, Claire Karekezi, Angelos Kolias, Carlo Serra, Luca Regli, Tiit Mathiesen, Victor E. Staartjes

**Affiliations:** 1 Machine Intelligence in Clinical Neuroscience & Microsurgical Neuroanatomy (MICN) Laboratory, Department of Neurosurgery, Clinical Neuroscience Center, University Hospital Zurich, University of Zurich, Zurich, Switzerland; 2 Department of Neurosurgery, The Royal London Hospital, Barts Health NHS Trust, London, UK; 3 Institution of Neuroscience & Physiology, Sahlgrenska Academy, Gothenburg, Sweden; 4 Art Clinic Hospitals, Gothenburg, Sweden; 5 Dept of Neurosurgery, Azienda Ospedaliero-Universitaria Senese, Siena, Italy; 6 Neurosurgery Unit, Department of Surgery, Rwanda Military Hospital, Kigali, Rwanda; 7 Division of Neurosurgery, Addenbrooke’s Hospital, University of Cambridge, Cambridge, UK; 8 University of Copenhagen, Copenhagen, Denmark; 9 Rigshospitalet, Copenhagen, Denmark; 10 Karolinska Institutet, Stockholm, Sweden; 11 Group Leader, MICN Laboratory, Department of Neurosurgery, University Hospital Zurich, Clinical Neuroscience Center, University of Zurich, Frauenklinikstrasse 10, Zurich, CH 8091 Switzerland

**Keywords:** Neurosurgery, Randomized controlled trial, Social media, Twitter, X

## Abstract

The importance of social media has seen a dramatic increase in recent times, but much about its influence in academia is still unknown. To date, no comparative studies analysing the effect of social media promotion on citation counts have been undertaken in neurosurgical publishing. We randomized 177 articles published in *Acta Neurochirurgica* from May to September 2020. The 89 articles in the intervention group received a standardized social media promotion through one post on our official Twitter/X account, whereas the 88 articles in the control group did not receive any social media promotion. Citation counts, website visits and PDF downloads were tracked at one and two years post-promotion. We found no significant difference in number of citations at one year post-promotion (Intervention: 1.85 ± 3.94 vs. Control: 2.67 ± 6.65, *p* = 0.322) or at two years (5.35 ± 7.39 vs. 7.09 ± 12.1, *p* = 0.249). Similarly, no difference was detected in website visits at one (587.46 ± 568.04 vs. 590.65 ± 636.25, *p* = 0.972) or two years (865.79 ± 855.80 vs. 896.31 ± 981.97, *p* = 0.826) and PDF downloads at one (183.40 ± 152.02 vs. 187.78 ± 199.01, *p* = 0.870) or two years (255.99 ± 218.97 vs. 260.97 ± 258.44, *p* = 0.890). In a randomized study, a structured promotion of general neurosurgical articles on Twitter/X did not significantly impact citation count, website visits, or PDF downloads compared to no social media promotion. Combined with published evidence to date, the impact of social media on citation counts in academic publishing ultimately remains unclear.

## Introduction

Over the past few years, the importance of social media in scientific publishing has risen massively. Nowadays, there are few respected scientific journals that do not operate at least one social media platform – may that be Twitter/X, Instagram, Facebook, or LinkedIn. Most journals use their social media profiles to promote selected articles that are published online, by summarizing their findings, showing illustrative figures from the publication, or by directly linking to the publication itself.

Little is known about the impact of social media coverage on audience reception, views/downloads, and citations of the promoted publications. Especially comparative studies are few and far between. Widmer at al. [1] conducted a randomized controlled trial (RCT) to study these effects for the journal “Mayo Clinical Proceedings”. Among the 68 randomized publications, the website access and download rate was significantly and relevantly higher whenever the publications were promoted via Twitter/X, Facebook, and LinkedIn. Similarly, Luc et al. [2] conducted a RCT and found that the 112 articles promoted via Twitter/X were cited significantly more after one year than the articles in the control group.

The effects of social media promotion of scientific articles have been studied in various fields of medicine [2–7]. We have previously shown that a structured social media campaign may increase article website visits and portable document format (PDF) downloads compared to historical controls [8]. There is, however, no published evidence on the comparative effects of social media promotion in neurosurgical publishing, especially not on citation counts. A potential influence of social media promotion – as a factor extrinsic to the merits of the study itself - on citations may relevantly bias the neurosurgical literature. For this reason, we carried out a RCT of article promotion on Twitter/X for publications in the neurosurgical general journal *Acta Neurochirurgica*, evaluating the effect on citation count at 1 and 2 years post-publication.

## Materials and methods

### Design

All articles published in the general neurosurgical journal *Acta Neurochirurgica* between May 6 and September 14, 2020 were randomly allocated to either a structured social media promotion, or to no social media promotion at all. Website visits, full-text PDF downloads, as well as citations were tracked for all included articles at 1 year and at 2 years post-promotion.

### Criteria for inclusion and exclusion

We included all non-invited articles published in *Acta Neurochirurgica* during the study period. All invited contributions were excluded. Letters to the editor and responses to letters, as well as editorials were also excluded.

### Randomization

Articles were allocated to the study groups by the social media editors using a pre-generated, stratified permuted block randomization sequence. Randomization occurred in random permutated blocks of 4, 6, and 8, with stratification according to open access status, Quarter of the Year (January-March; April-June; July-September; October-December), and Review Status (Review Paper vs. Original Article), since review articles and those published open access have a tendency towards higher citation rates [9, 10]. Randomization and subsequent interventions were carried out immediately after inclusion.

### Intervention

Articles allocated to the intervention group received one post on the official *Acta Neurochirurgica* Twitter/X account, containing the paper title, standardized hashtags (#), a link to the journal’s online publication of the respective article, an interesting figure or table from the article if available, and the flags of the country of origin of the article. The promotion took place at the time of online first publication (as opposed to journal issue inclusion). The standardized hashtags included: #nsgy, #neurosurgery, #SoMe4Surgery, #OnlineFirst, and #OpenAccess or #EditorsChoice when applicable. If identifiable, the primary and/or senior authors or the authors’ institutional Twitter/X account were tagged. The social media editors of *Acta Neurochirurgica* reposted the standardized promotion on their personal Twitter/X accounts, and partly added a brief explanatory sentence.

### Outcome measures

The primary endpoint was the number of citations on Google Scholar at 2 years after the structured promotion. Citations at 1 year post-promotion were also tracked. As secondary endpoints, the number of website visits and full-text PDF downloads were tracked at 1 year and 2 years post-promotion. The number of full-text PDF downloads of each included article was kindly provided by Springer Nature.

### Sample size calculation

At the time of study planning, on average, 2-year citations for Acta Neurochirurgica, based on Journal Citation Reports (Citations in 2016 and 2017, Clarivate Analytics), amounted to 2.65 ± 2.35. To demonstrate an average difference of 1 citation (2.0 citations expected in the control group vs. 3.0 citations expected in the intervention group) between the two groups, with a statistical power of 1 – β = 0.8 and a Type I error rate of α = 0.05, it was determined that a minimum sample size of 88 articles needed to be included per group.

### Statistical analysis

Continuous variables were given as means ± standard deviations (SD), and categorical variables as numbers (percentages). Analyses were carried out following the intention-to-treat principle. Citation counts are not normally distributed but follow a Poisson distribution, therefore independent t-tests were used to compare the primary and secondary endpoints among the two study groups. A *p* ≤ 0.05 on two-tailed tests was regarded as statistically significant. R Version 4.1.1 was used for the analyses [11]. The CONSORT statement was implemented [12]. 

## Results

**Fig. 1:**
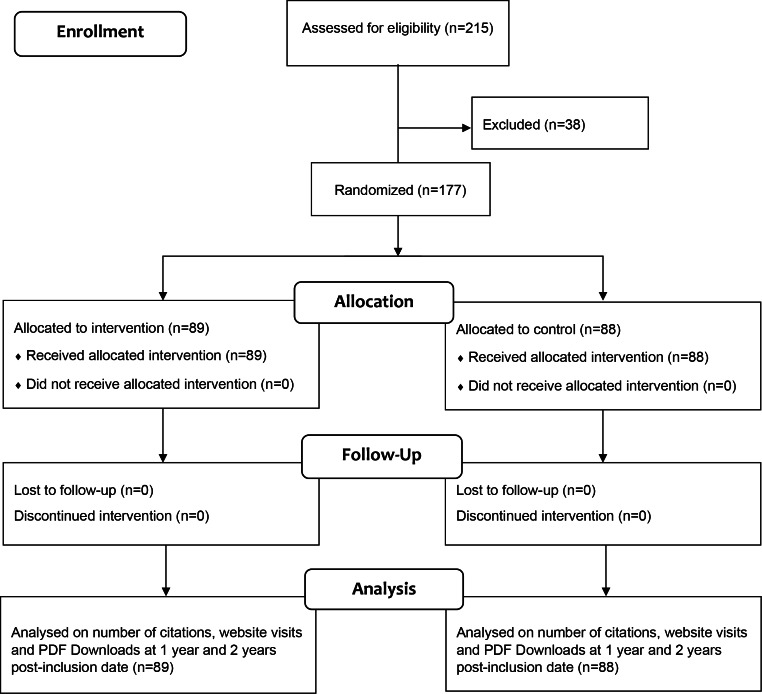
Study flow diagram detailing article randomization and allocation to the social media intervention group and control group

We included 177 consecutive eligible articles of the 215 articles published in *Acta Neurochirurgica* in the period of May 6 to September 14, 2020. Of the included articles, 89 papers were allocated to the intervention group, while 88 papers were allocated to the control group (Fig. 1). All articles were able to be followed-up until completion of the study, 2 years post-inclusion of the final randomized article. Characteristics of the included articles are detailed in Table 1.

**Table 1:** Baseline characteristics of included articles

	Overall	Intervention Group	Control Group
**Number of Papers**,** n (%)**	177 (100%)	89 (50.3%)	88 (49.7%)
**Type of Article**,** n (%)**			
Original Article	112 (63.3%)	57 (64.0%)	55 (62.5%)
Case Report	19 (10.7%)	9 (10.1%)	10 (11.4%)
How I do it	23 (13.0%)	12 (13.5%)	11 (12.5%)
Review Article	14 (7.9%)	7 (7.9%)	7 (8.0%)
Technical Note	9 (5.1%)	4 (4.5%)	5 (5.7%)
**Editor’s Choice**,** n (%)**	1 (0.6%)	1 (1.1%)	0 (0%)
**Open Access**,** n (%)**	49 (27.7%)	25 (28.1%)	24 (27.2%)
**Author in Tweet**,** n (%)**	-	16 (18.0%)	-
**Figure in Tweet**,** n (%)**	-	85 (95.5%)	-

### Primary endpoint: Citation Count

**Fig. 2:**
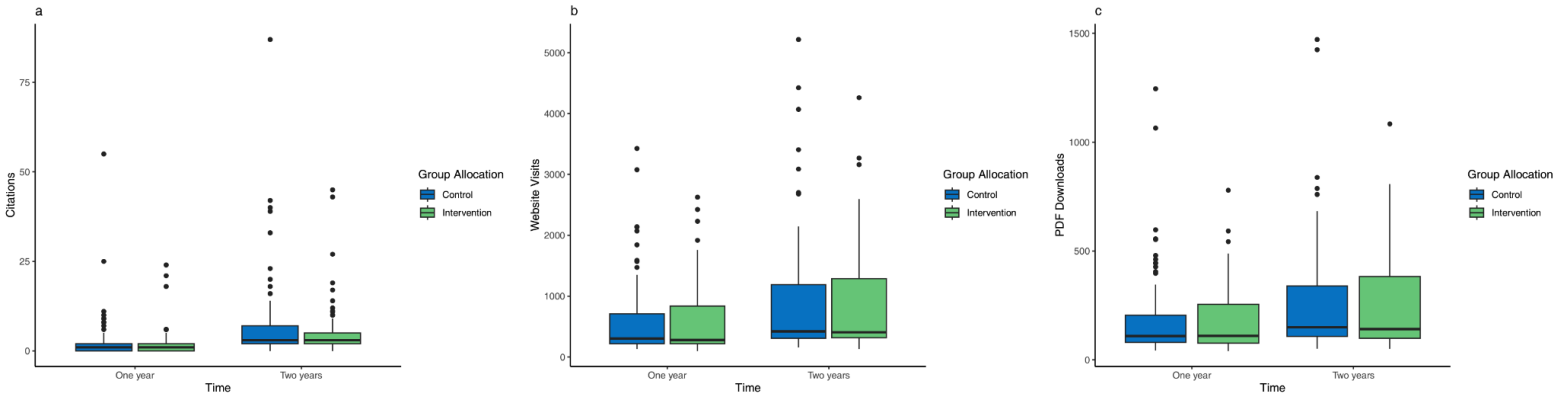
Boxplots illustrating the range of citations (**a**), website visits (**b**), and PDF downloads (**c**) for the intervention and control group at one year and two years post-inclusion

We found no statistically significant difference (Table 2) in number of citations between the intervention and control group neither at 1 year post-promotion (Intervention: 1.85 ± 3.94 vs. Control: 2.67 ± 6.65, *p* = 0.322), nor at 2 years (Intervention: 5.35 ± 7.39 vs. Control: 7.09 ± 12.1, *p* = 0.249, Fig. 2).

**Table 2:** Summary of results after randomization into social media promotion (intervention) or no promotion (control) groups during the study period. Citation counts were derived from Google Scholar. Means ± standard deviations are provided

	Intervention	Control	*p*
**Citation Count**			
1 year	1.85 ± 3.94	2.67 ± 6.65	0.322
2 years	5.35 ± 7.39	7.09 ± 12.1	0.249
**Website Visits**			
1 year	587.46 ± 568.04	590.65 ± 636.25	0.972
2 years	865.79 ± 855.80	896.31 ± 981.97	0.826
**PDF Downloads**			
1 year	183.40 ± 152.02	187.78 ± 199.01	0.870
2 years	255.99 ± 218.97	260.97 ± 258.44	0.890

### Secondary endpoints: website visits and PDF downloads

No statistically significant difference was detected between groups in website visits at 1 year (Intervention: 587.46 ± 568.04 vs. Control: 590.65 ± 636.25, *p* = 0.972) and 2 years (Intervention: 865.79 ± 855.80 vs. Control: 896.31 ± 981.97, *p* = 0.826). Similarly, the number of PDF downloads at 1 year (Intervention: 183.40 ± 152.02 vs. Control: 187.78 ± 199.01, *p* = 0.870) and at 2 years (Intervention: 255.99 ± 218.97 vs. Control: 260.97 ± 258.44, *p* = 0.890) post-promotion was not significantly different.

## Discussion

In this study, articles published in *Acta Neurochirurgica* were randomized to either receive a standardized promotion through a single post on Twitter/X or to receive no promotion on social media. Number of citations, website visits, and PDF downloads after 1 and 2 years post-promotion were collected and analysed. We found that there was no significant difference in all outcome measures between the articles that had undergone social media promotion compared to the ones which had not, neither at 1 nor at 2 years after the intervention.

With the rising presence of social media in the past years, interest into its potentially increasing influence has grown. However, there remains no clear consensus on the effect of social media on academic medical publishing. In a previous study, we had compared PDF downloads and website visits within the first year of our social media campaign with historical controls from the year before social media promotion had started [8]. In that study, we had found no overall difference in website visits and PDF downloads between the historical control period without promotion, and the first year of the social media campaign. However, within a subgroup analysis, promoted articles appeared to be significantly more frequently viewed and downloaded than non-promoted ones. These results do not necessarily reflect the currently presented data from this RCT but are potentially explained by the current data stemming from randomized group allocation – whereas in the previous study, specific and potentially more interesting articles had been hand-picked by the editorial team. Taking these data together, it thus appears as if unselected promotion may not necessarily lead to a measurable impact, but that selective promotion of highly interesting articles may be more effective.

Our current study appears to be the first dealing with citation counts and the first to perform a controlled study on the effects of social media in neurosurgical publishing. In other fields, however, there have been several RCTs, which have shown more favourable outcomes after social media promotion. Widmer et al. [1] reported a higher rate of website visits and full-text PDF downloads after 30 days in articles that had received a social media promotion compared to articles that had not. Their promotion strategy consisted of 7 posts per article distributed evenly across various accounts on Twitter/X, Facebook, and LinkedIn. They do, however, specify that this increase in traffic did not result in a higher number of citations. In contrast, Luc et al. [2] have found a significantly higher citation count after 1 year in articles exposed to social media, where the articles were randomized and promoted on the personal Twitter/X account of a designated delegate and subsequently retweeted by 11 further delegates with a combined following of 52’983 individuals. Similarly, Ladeiras-Lopes et al. [5] have most recently found a positive impact of social media promotion on citation count 2 years after intervention in a RCT. Still, some other RCTs have suggested similar findings to our own: Fox et al. [13] have found no increase in website views of promoted articles 30 days after intervention, where articles were posted on Facebook and Twitter/X. By the end of the study, they reported a following of 28,177 and 2219 on Facebook and Twitter/X respectively. In a follow up study an increased intensity of the strategy, where focus was laid on gaining more followers and increasing frequency of posts per article, did not cause an increase in website views in the promoted articles either [14]. Similarly, Tonia et al. [15, 16] found no significant advantage to promoted articles in terms of citation count, website views, or PDF downloads, using a strategy where articles were promoted once on the journal’s blog, as well as twice on both Twitter/X and Facebook.

In social media promotion in academic publishing, there are several important factors to be considered. The number of followers on social media could have an impact on the success of a promotion strategy, as well as the platforms employed. More followers equate to better dissemination and further reach, which in itself could have a relevant impact [17, 18]. Furthermore, the implementation of various mediums of social media (e.g. mentions on Twitter/X, dissemination through podcasts or blog posts) could further impact the success of a social media strategy [19, 20]. A higher intensity of promotion (e.g. frequent posts pertaining to one article or posting on several social media platforms) could similarly be a relevant factor [17, 21]. Although, as mentioned, it has been suggested that this may not be the case, at least concerning an increased frequency of posts regarding one article [14]. Intuitively, the usage of several social media platforms may also be considered as a potentially relevant factor in increasing reach, though there is research yet to be conducted in properly examining this potential connection. Using different platforms compared to traditional social medias, however, could be of advantage, as proposed by Kudlow et al. [22, 23], who promoted articles on a cross-publishing article recommendation and distribution platform and found a relevant impact on citation count. Moreover, it has been suggested that promotion on personal accounts rather than official accounts may have a higher social media impact [24]. 

While it intuitively seems plausible that social media promotion may increase article reach, any potential influence of social media promotion on citation counts down the line may be seen as problematic. Studies should be evaluated and selected for citation through critical appraisal of their methodology, underlying data, clinical impact and applicability, and proper acknowledgment of their limitations. In other words, confounding factors such as potentially social media promotion ought not to bias the way that studies are selected for citation or for systematic reviews, but rather they ought to be appraised for their scientific merit. Most of the published evidence including our current study does not suggest any or at least not any major bias in citation counts after social media promotion [1, 15, 16]. In any case, our data appear to corroborate that unselected social media promotion may not be nearly as impactful as highly selective, targeted promotion of high-impact articles.

### Strengths and limitations

Our study followed a rigorous methodology and randomization resulted in two highly comparable group, allowing for a statistically powerful pre-planned analysis. Follower count was not collected at the time of intervention. At the time of analysis, the follower count was 3471 (accessed on July 21, 2023). It must be noted that because of this, follower count cannot be correctly compared to the other studies. However, considering that *Acta Neurochirurgica* joined Twitter/X in April 2019 and the intervention began one year later, it can be assumed that follower count was significantly lower at the time of intervention. It stands to reason that a lower follower count could be a relevant factor to keep in mind in the interpretation of our results, and that the effect of promotion may show a strong “dose-effect” correlation. With that in mind, our results may not necessarily be extrapolated to cases with higher follower count. Similarly, impact factor of the journal may be a relevant factor in this ongoing discussion and our results may therefore not be generalized to cases with higher impact journals. While the goal of this study was to analyze the effects of social media promotion on neurosurgical publishing, it ought to be considered that our findings may not be applicable to academic publishing in other fields of medicine. We conducted a structured promotion of unselected articles and therefore our study does not take into account expected impact of the articles or the citability of authors (quantified by h-indices or the number of previous publications). Similarly, the time of posts as well as number of shares and retweets were not considered in this study. It is conceivable that these variables could influence the performance of the social media promotion. Our intervention was clearly defined and is reproducible, as it consisted of only one post on Twitter/X. This is a less intense promotion strategy than in some other studies. It could be argued that a higher intensity of promotion through more frequent posts and the use of several social media platforms may more strongly impact reach and therewith potentially citations.

## Conclusion

In a randomized study, a structured promotion of general neurosurgical articles on Twitter/X did not significantly impact citation count, website visits, or PDF downloads, compared to no social media promotion. The relationship between social media promotion and article performance is complex and may be subject to a multitude of factors. In combination with the published evidence from other medical domains, the impact of social media on citation counts in academic publishing ultimately remains unclear.

## Data Availability

The data in support of our findings can be obtained upon reasonable request from the corresponding author.
